# Intrathoracic Kidney after Blunt Abdominal Trauma: A Case Report and Review of the Literature

**DOI:** 10.1155/2015/682649

**Published:** 2015-12-31

**Authors:** Fikret Halis, Akin Soner Amasyali, Aysel Yucak, Turan Yildiz, Ahmet Gokce

**Affiliations:** ^1^Department of Urology, School of Medicine, Sakarya University, 54100 Sakarya, Turkey; ^2^Department of Urology, School of Medicine, Adnan Menderes University, 09000 Aydın, Turkey; ^3^Department of Pediatric Surgery, School of Medicine, Sakarya University, 54100 Sakarya, Turkey

## Abstract

Abdominal trauma is responsible for most genitourinary injuries. The incidence of renal artery injury and intrathoracic kidney is quite low in patients who present with blunt trauma experiencing damage. There are four defined etiologies for intrathoracic kidney, which include real intrathoracic ectopic kidney, eventration of the diaphragm, congenital diaphragmatic herniation, and traumatic diaphragmatic rupture. The traumatic intrathoracic kidney is an extremely rare case. We presented intrathoracic kidney case after traumatic posterior diaphragmatic rupture.

## 1. Introduction

The kidney is the most commonly injured organ after genitourinary (GU) trauma, with renal injury occurring in almost half of cases. In the pediatric population, renal injury occurs in 10–20% of all abdominal blunt trauma and 3-4% of penetrating trauma [1]. Furthermore, patients experience injury to other organs in 42–74% of GU trauma cases. These injuries include colonic perforation, as well as vertebral and rib fractures, which can lead to renal lacerations because of direct penetrating injury [2].

Renal trauma is observed more often in children than adults. Children are more prone to renal trauma because they have relatively larger kidneys located lower in the abdomen. In addition, the abdominal wall of children is weaker in comparison to adults [2–4]. Moreover, the risk of lower pole and parenchymal injury in children is increased because their kidneys are lobulated. Finally, because of increased mobility in the pediatric kidney, renal pedicle and ureteropelvic junction injuries are more common [4]. Damage to the kidney from GU trauma can range from a simple laceration to pedicle splitting. We present a case of an intrathoracic kidney after blunt abdominal trauma.

## 2. Case Presentation

A twelve-year-old boy was redirected to the emergency department after being involved in a motor vehicle accident. Upon arrival, a blood transfusion was given, and urethral catheter was inserted, revealing macroscopic haematuria. While taking history, we learned that a vehicle passed over the patient. The physical examination revealed hypotension (90/50 mmHg), tachycardia (140/min), and decreased partial O_2_ pressure (SPO_2_ = 90). Pelvic pain and a subcutaneous hematoma were detected. Furthermore, abdominal pain and guarding were observed during palpation. Auscultation revealed absent breath sounds over the left chest.

**Figure 1:**
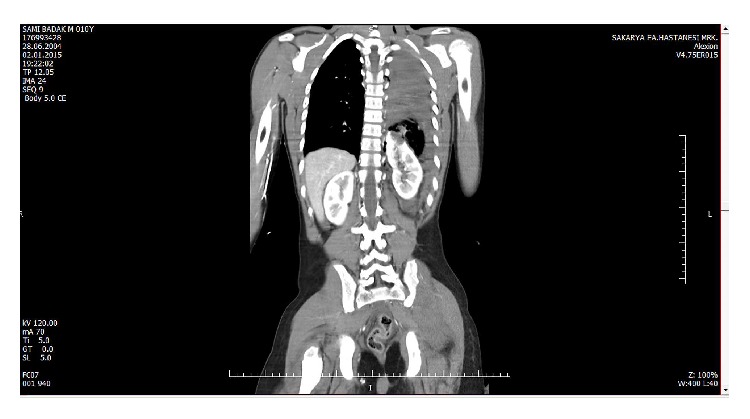
CT scan imaging of left diaphragmatic rupture and left intrathoracic kidney.

Laboratory analysis revealed a WBC of 24000/*μ*L and a Hb of 7 g/dL. Other laboratory parameters were as follows: urea, 55 mg/dL; creatinine, 1.6 mg/dL; AST, 242 U/L; and ALT, 158 U/L. A left-sided pelvic fracture was detected in pelvic X-ray. Chest X-ray revealed a left hemopneumothorax and two costal fractures. CT scan confirmed the hemopneumothorax and revealed a left diaphragmatic rupture and left kidney herniation (Figure 1). In addition, a laceration to the spleen was observed with widespread fluid collection in the abdomen.

During stabilization, a blood transfusion was given in the emergency department. A chest tube was inserted in order to relax breathing. Stabilization of vital signs was achieved by replacing the patient's fluid deficit. An exploratory laparotomy was performed for the diaphragmatic rupture in the fourth hour of admission. The operation revealed a collection of blood and a herniated left kidney through the posterior diaphragm, as seen on CT scan. There was no observable trauma to the renal pedicle. The kidney was repositioned to normal location, and the ureteral rupture was controlled by contrast ureteropyelography under fluoroscopic guidance. No urinary extravasation was detected along the urinary system, from the ureteropelvic junction to the bladder. Subsequently, the diaphragmatic defect was repaired. Postoperative follow-up revealed normalization of clinical parameters.

## 3. Discussion

Abdominal trauma is responsible for 90% of GU injury. Specifically, renal injury is seen in 10–20% of all abdominal blunt trauma [2]. The incidence of renal artery injury is quite low, with only 0.08% of patients who present with blunt trauma experiencing damage [5]. The intrathoracic kidney is a rare pathologic condition with four defined etiologies, which include real intrathoracic ectopic kidney, eventration of the diaphragm, congenital diaphragmatic herniation, and traumatic diaphragmatic rupture [6]. The traumatic intrathoracic kidney is an extremely rare case. We presented intrathoracic kidney case after traumatic posterior diaphragmatic rupture.

Traffic accidents, sports injuries, falls, and assaults are the main causes of blunt renal trauma in children [7]. Revised renal injury classification was described by American Association for the Surgery of Trauma: grade 1, subcapsular hematoma; grade 2, laceration < 1 cm in depth into cortex; grade 3, laceration > 1 cm in depth into medulla; grade 4, laceration through the corticomedullary junction into the urine-collecting system or segmental renal vascular injury with haematoma; grade 5, shattered kidney [8]. Although macrohaematuria is a common sign of GU trauma, it does not predict the severity of the injury. In fact, haematuria suggests major renal injury in only 25% of cases [7]. Ruptured arcuate and interlobular veins are the most common reason of haematuria in cases of minor trauma [9]. In our case, macrohaematuria might have occurred from the rupture of these veins or the mucosal and submucosal distention of the pelvicalyceal system. However, there was no evidence of major renal vascular injury. Shock is another presenting sign of severe renal injury. However it is not a reliable indicator for children, because it occurs in only 5% of cases. Furthermore, even when hypotension and shock do occur in children, they are often not clinically observed within the first hours of trauma [7].

Shattered major renal segments are more likely to occur in children due to the mobility of their kidneys. But in our case, despite the intrathoracic displacement of the kidney, the renal pedicle and ureteropelvic junction were normal. Herniating with its pedicle might have prevented the rupture of major renal segments. There are few case reports about traumatic intrathoracic kidney in the literature. In one particular case report, Esquis et al. noticed intrathoracic herniation of the left kidney 20 years after the initial motor vehicle accident and subsequently reduced the kidney back within the abdomen and closed the hiatus using a laparoscopic approach [10]. Pascual Samaniego and colleagues reported another case of a traumatic intrathoracic kidney and suggested that the rise in intra-abdominal pressure causes herniation of the kidney through a preexistent congenital pathway [11].

A contrast-enhanced CT scan is indicated for all children with blunt renal injury. Grading the renal injury with CT scan determines the management [12]. Usually after the inciting trauma, the herniated kidney is localized to the posterior mediastinum. It therefore may mimic posterior mediastinal masses, such as a Bochdalek hernia, neurogenic mass, or sequestration [6, 13]. However, the typical appearances of the kidney, such as the enhanced pelvicalyceal system, can easily be seen with a contrast-enhanced CT scan, thus allowing us to differentiate the kidney from other structures.

A left-sided, herniated kidney is more common in comparison to the right [14], as in our case. Left hemidiaphragm congenitally has a weaker structure than the right side, and the liver serves as extra protection for right side; that is why trauma is more likely to rupture the left diaphragm especially in children [15]. However, Inokuchi et al. reported a right intrathoracic kidney after blunt abdominal trauma [16]. The patient in their review was a 67-year-old man and died from haemothorax after being run over by a low-speed vehicle. The authors diagnosed the herniated kidney after autopsy, revealing that the right kidney had passed into the intrathoracic space. Age, occurrence of trauma, and previous surgeries might affect the herniation side. They stated that the haemothorax might have originated from the retroperitoneum after the blunt abdominal trauma.

Treatment of an intrathoracic kidney should include reduction of kidney, followed by repair of the ruptured diaphragm, using either open or laparoscopic approach. Urinary extravasation should be evaluated by contrast ureteropyelography under fluoroscopic guidance. Serial hemogram, physical examination, and blood pressure monitoring should also be performed following surgical intervention. Three days postoperatively a control CT scan may prove to be beneficial in assessing posttraumatic changes, as well as the need for further intervention.

In conclusion, although it is a rare condition, diaphragmatic rupture and kidney herniation should remain a part of the differential diagnosis when a mass is seen in posterior mediastinum on the CT scan after blunt trauma.
